# Inter-Rater Reliability of the Polish Version of the Alberta Infant Motor Scale in Children with Heart Disease

**DOI:** 10.3390/jcm12134555

**Published:** 2023-07-07

**Authors:** Maria Ferenstein, Katarzyna Ostrzyżek-Przeździecka, Jakub S. Gąsior, Bożena Werner

**Affiliations:** Department of Pediatric Cardiology and General Pediatrics, Medical University of Warsaw, 02-091 Warsaw, Poland

**Keywords:** Alberta Infant Motor Scale, motor development, children, heart disease, inter-rater reliability, agreement

## Abstract

There is an urgent need for the systematic monitoring of motor and cognitive neurodevelopment and the evaluation of motor skill development in infants and children with heart disease. Familiarizing students and early graduates with the developmental care needed by these patients may help in the system-wide implementation of early motor screening in this population. The purpose of this study was to investigate the agreement between a last-year physiotherapy student and an experienced pediatric physiotherapist when applying the Polish version of the Alberta Infant Motor Scale (AIMS) to a heterogenous group of children with congenital heart defects. Agreement between raters was verified based on the observation of 80 (38 females) patients with heart disease aged 1–18 months using a Bland–Altman plot with limits of agreement and an intraclass correlation coefficient. The bias between raters for the total score for four age groups (0–3 months, 4–7 months, 8–11 months and 12–18 months) was between −0.17 and 0.22 (range: −0.54–0.78), and the ICC was between 0.875 and 1.000. Thus, a reliable assessment of motor development or motor skills using the Polish version of the AIMS can be performed in pediatric patients with heart defects by clinically inexperienced last-year physiotherapy students who are familiarized with the AIMS manual.

## 1. Introduction

An increasing number of studies have reported delayed motor milestone achievements, the impaired quality of motor behavior and general motor problems in patients with complex congenital heart disease (ConHD) [1,2,3,4,5,6,7,8,9,10,11,12,13,14,15,16,17]. Indeed, children with ConHD have an 11-fold risk of developing severe motor problems compared to their peers without any known heart failure [18]. It has been shown that the poorer motor development outcomes from infancy to adolescence in this population are related to various modifiable and non-modifiable factors: the presence of genetic anomalies [19,20,21,22,23]; alterations in brain development due to brain injury [7,11,24,25]; a low birth weight [21,26]; single-ventricle physiology/anatomy [8,14,16,27,28,29,30]; surgery in early infancy [31]; a higher number of cardiac surgeries [20,29,32,33,34]; palliative surgery intervention(s) [34,35]; pre-, peri- and post-operative complications (e.g., preoperative hypoxemia [36,37,38]; perioperative white matter injury [39]; a long intubation period [40]; a long hospital stay [5,20,29,40,41,42,43,44,45,46]; and others like the need for tube feeding [22,29] or being of the female sex [43]).

Authors of recent articles on motor development delay and lower basic motor skills in patients with ConHD stressed the importance of early motor screening in infants, but also indicated the need for the systematic longitudinal evaluation and monitoring of motor skill development through the elementary school years [3,6,8,9,13,14,16]. Very recently (2023), significant gaps in the research on developmental care for hospitalized infants with complex ConHD were identified by the American Heart Association Science Advisory Group—one of them was to understand how to measure and promote gross motor development in infants with complex ConHD [9].

Valid, reliable, convenient and relatively inexpensive scales for assessing motor development and motor competence are necessary to allow wide group of researchers and practitioners to identify motor development deficiencies in pediatric cardiac patients and to determine the effectiveness of selected interventions. The scales for evaluating motor development such as, e.g., the Bayley Scales of Infant Development, require specific training and usually have a high cost [47]. The Alberta Infant Motor Scale (AIMS) is an easy and fast-to-apply standardized observational assessment scale providing information on achieved developmental milestones, currently developing motor skills/activities and motor performance that is widely used in term and preterm infants from birth until they achieve independent walking [48]. The construction of the scale makes it approachable for both novice and experienced researchers [49]. High agreement between groups of rater/specialists with the same (experienced and/or trained pediatric physical therapists, early intervention specialists with a minimum of three years of experience, and physiotherapists, neurologists and physiatrists) or a different experience in the evaluation of pediatric patients (trained students vs. experienced examiners or physical therapists with different levels of experience) using the AIMS based on live or video observations of typically developing and preterm infants, as well as those at risk for motor delay, was confirmed [48,49,50,51,52,53,54,55]. Satisfactory agreement between experienced, trained specialists was also found with different versions of the AIMS other than the English language version [56,57,58,59,60,61]. In 2022, cultural adaptation and validation of the Polish version of the AIMS were assessed. The reliability of the observation of the developmental assessment of apparently healthy infants between two experienced (minimum 7 years) pediatric physiotherapists was 0.99 [62]. Very recently (2023), the authors established the norms of the Polish AIMS version [63].

In infants with heart disease, there is an urgent need to monitor motor development systematically and, in cases of developmental delay, to promote early intervention as soon as possible to harness neuroplasticity [8]. Motor development in children with heart defects was previously assessed according to the AIMS; however, it was only carried out by experienced/trained specialists [6,11,64]. The observation of motor development using standardized measures is a core competence in pediatric physical therapy education. Familiarizing students and early graduates with the developmental care needs of hospitalized infants with complex heart disease may help in the dissemination and implementation of early motor development monitoring in this population. However, early-stage practitioners may under- or over-report the number of children with motor developmental delay when using selected tools or scales; therefore, agreement between more experienced clinicians and students when performing the assessment should be primarily verified. The purpose of this study was to investigate agreement between two raters—a last-year physiotherapy student and pediatric physiotherapist with 10 years of clinical experience—with the Polish version of the AIMS in a heterogenous group of children with congenital heart defects.

## 2. Materials and Methods

### 2.1. Population

The study was conducted at the Department of Pediatric Cardiology and General Pediatrics of the Medical University of Warsaw. Infants aged 0 to 18 months with a diagnosed heart disease qualified for the study. The exclusion criteria were: less than six weeks since last sternotomy surgery, central venous cannula, and non-invasive ventilation. Demographic and clinical data about infants were collected in terms of: sex, gestational age, birth weight, mode of delivery, Apgar score, and type of heart anomaly. This study is a part of the research project on the assessment of determinants of motor development and physical activity levels in pediatric patients with heart disease.

### 2.2. Raters and Assessment

Two physiotherapists examined the patients simultaneously: the first one has a higher master’s degree in physiotherapy and 10 years of experience in working with toddlers and the AIMS (experienced assessor—K.O-P.), and the second is a last-year (5th-year) physiotherapy student interested in pediatric physical therapy (inexperienced assessor—M.F.). The student did not undergo professional AIMS training, but she was encouraged to be familiar with the AIMS manual (English language) before the start of the examinations [65]. During the examinations, both observers performed a motor assessment without consulting with each other’s observations during the course of the study.

The assessment was performed according to the manual [65]. The parent/caregiver was informed that the motor assessment was to be conducted during the second day of hospitalization and was asked about the time of day when the child is awake, with a time chosen at least 30 min after their last meal and when they would be active. The parent/caregiver was present during the assessment and instructed to interact with the child to achieve and maintain an optimal state. If the child was anxious, both examiners observed their motor abilities from a distance and guided the parent/caregiver on how to interact with the child.

### 2.3. Assessment Tool

The assessment tool used was the Polish language version of the Albert Infant Motor Scale [62,63,66]. The Polish version of the AIMS was recently validated and adapted for infants aged 0–18 months and can be applied to this population for clinical and scientific purposes [62]. The Alberta Infant Motor Scale is a norm-referenced, observational scale that evaluates the gross motor development of children from birth to 18 months, until the achievement of independent walking. The scale contains 58 items organized into four subscales describing the development of spontaneous motor skills in four positions: prone (21 items), supine (9 items), sitting (12 items) and standing (16 items) [48,65,66]. The examiner observes the movement of the infant in each one of the positions with minimal handling, taking into account weight bearing, posture and antigravity movements. Gross motor development is defined by the AIMS total score, which is expressed in percentiles based on age. Cut-off points at the 10th centile at 4 months and the 5th centile at 8 months based upon the normative data have been shown to be predictive of abnormal development [48,65,66]. Depending on the patient’s age, the assessment was performed in a crib or on a mat. Each infant assessment took between 10 to 20 min depending on infant age [48,65,66].

### 2.4. Statistical Analysis

Agreement on the AIMS score between assessors was verified using a Bland–Altman plot with limits of agreement (LoA) [67] and an intraclass correlation coefficient (ICC) [68], and interpretation was carried out using that proposed by Hopkins et al.: 0 to 0.30, small; 0.31 to 0.49, moderate; 0.50 to 0.69, large; 0.70 to 0.89, very large; and 0.90 to 1.00, nearly perfect [69]. All calculations were performed using STATISTICA 13 (StatSoft Inc., Tulsa, OK, USA) and MedCalc software version 19.4.1 (MedCalc Software, Ostend, Belgium). The Bland–Altman plots were created using PQStat version 1.8.4 (Poznań, Poland). GraphPad Prism 5 (GraphPad Software Inc., San Diego, CA, USA, 2005) was used to create a correlation plot.

## 3. Results

Of the 80 (n = 38 females) pediatric patients with heart disease (ConHD, n = 75; cardiac arrhythmia, n = 3; and cardiomyopathy, n = 2) who participated in the study, 38 were born vaginally and 42 were born by cesarean delivery. Participants’ median age, median gestational age, median birth weight and median Apgar score were 7 months (range: 1–18), 39 weeks (range: 27–41), 3200 g (range: 840–4490) and 10 points (range: 5–10). Patients’ characteristics by four age groups (0–3 months, 4–7 months, 8–11 months, and 12–18 months) are presented in Table 1.

**Figure 1:**
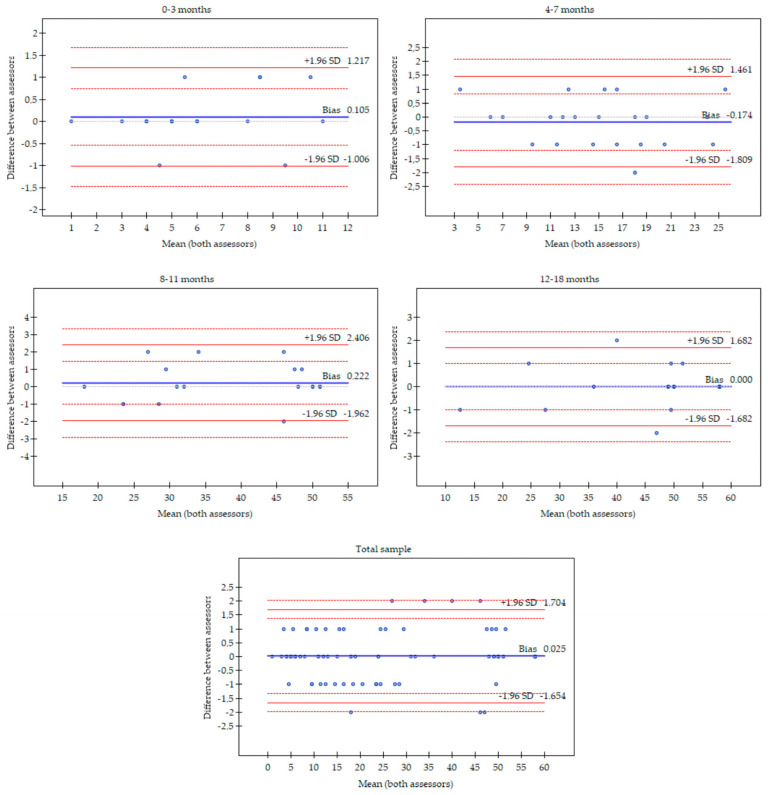
Bland–Altman plots representing the agreement between experienced and inexperienced assessors in the AIMS total score for selected age groups and total sample. The solid blue line indicates the bias, solid red lines are the 95% LoA (±1.96 SD) and dotted lines are confidence interval LoA.

**Figure 2:**
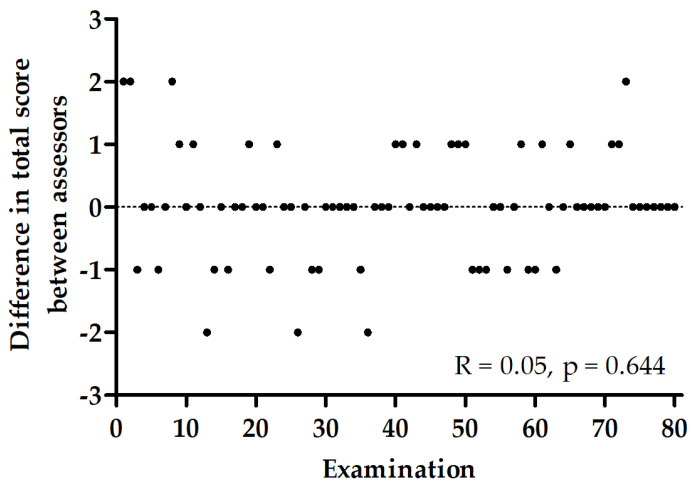
Correlation between difference in total score for the total sample between assessors and number of examinations. R—Spearman correlation coefficient.

The agreement statistics results are presented in Table 2. The lowest ICC values were observed for the youngest participants (0–3 months and 4–7 months) in a standing position. The highest bias for the AIMS total score was observed for participants aged 8–11 months (0.22), 4–7 months (−0.17) and 0–3 months (0.11). The Bland–Altman plots are shown in Figure 1, representing the agreement between experienced and inexperienced assessors in the AIMS total score for selected age groups and the total sample. There was no association between the number of examinations and differences between assessors in the AIMS total score for the total sample (Figure 2).

## 4. Discussion

The aim of this study was to analyze the inter-rater reliability of the Polish version of the AIMS in the pediatric population with heart disease based on two raters with different clinical experience. The results support high degrees of agreement between the raters for the total scores in each age group of patients and underscore that the assessment of motor development and/or motor skills using the AIMS is not dependent on the clinical experience of the observers. Motor development assessment using the AIMS can be performed in pediatric patients with heart defects aged 0–18 months by clinically inexperienced last-year physiotherapy students who are familiarized with the AIMS manual. Providing practical instructions (with resources) and guidance on how to measure and promote gross motor development in infants with complex heart defects to physiotherapy students and early-stage physiotherapists may help with filling in the gap in the research on developmental care for pediatric cardiac patients.

The results of the reliability and agreement statistics presented here are consistent with those from a healthy population from Canada [48] and Poland [62] (total ICC values of inter-rater reliability in both studies were also set at 0.99). Nevertheless, it is crucial to assess the inter-rater reliability of motor development scales in a diverse group of pediatric patients. Authors from China [61], Serbia [58] and Spain [59] have evaluated the inter-rater reliability of the AIMS among children at risk for delayed motor development (low birth weight, prematurity, low Apgar score, intraventricular hemorrhage, and neurological symptoms). In all of these studies, the ICC value for the total score was above 0.9 which proves the excellent reliability of the tool [58,59,61]. Taking into account these results presented here for children with heart defects, it can be concluded that the AIMS has high reliability not only among healthy children and can be used in infants with various types of disorders.

A tool that is minimally invasive, easy to use and culturally adaptable is needed to assess the motor development of children with heart defects. Albuquerque et al. examined the concurrent validity of the AIMS in relation to the gross motor subtest of the Bayley Scale III [47]. Delayed gross motor development was observed in 21% of patients according to the Bayley III, and in 12% for the 5th percentile and 21% for the 10th percentile of the AIMS. Adopting the AIMS 10th percentile as a cutoff point yielded the best combination of values for sensitivity and specificity for detecting motor delays [47]. The AIMS, unlike the Bayley III, allows for verification of weight bearing, posture and antigravity movements, which is helpful in the early detection of possible abnormalities. Some of the biggest advantages of the AIMS are that the examination requires minimal handling by the therapist and takes a maximum of 20 min, minimizing the risk of stress among high-risk children like those with heart defects. Despite its advantages, the AIMS has a couple of inconsistencies. One of them is the situation when the item “hands to feet” is observed, but the item “hands to knees” belonging to the “motor window” is not observed [51]. Raters are aware that if an infant is able to touch their feet, they should also be able to touch their knees; however, the latter item cannot be credited if it was not observed. This problem was described by Blanchard et al. [51], who decided to directly contact the co-author of the AIMS. Dr. Darrah recommended “not starting the window too far back and believes that experienced clinicians may have an advantage over less experienced clinicians in making this decision. When suspecting that an infant is capable of higher items, she recommends giving him or her time to “warm up” a little first before scoring.” [51]. However, this information is not included in the scale manual. According to the results and Dr. Darrah’s commentary, Blanchard et al. suggested that for infants younger than seven months of age, the evaluation in the supine position is more challenging to score reliably between raters than the other positions [51]. In our study, the ICC for the supine position among children aged 4–7 months was 0.986, which is one of the lower values compared to the other results. However, this value is still excellent, which emphasizes that the experience did not affect the motor evaluation of children with heart defects, and the author’s comment does not solve the problem.

When the child has mastered the ability to roll from a supine to a prone position with rotation, all items on the AIMS supine subscale can be considered as observed. Blanchard et al. pointed out that this item is dependent on the examiner’s ability to detect rotation in motion [51]. Misdiagnosis of rotation can falsify the supine subscale score in infants aged 4–7 months. In our study, both examiners detected rotation during rolling from supine to prone, and the ICC score for supine in this age group was 0.986. The lowest value of the ICC (0.875) was recorded for standing positions in the 0–3 month age group, which is consistent with studies conducted by the authors from Taiwan [53] and the Republic of Korea [57]. This correlation can be attributed to the low variety in standing positions (subjective assessment of active trunk control while standing) described (narrow range of results) for children in the first months of life. The examiner, while holding the child, has to independently decide on how to score the child’s movements in the standing position, which can cause discrepancies. In addition, in the first months of life, infants learn to control and lift their heads against gravity. The AIMS positions assume that the child can only lift their head in a pronated position to angles of 45 or 90 degrees, which significantly narrows the assessment by the examiner. According to Wang et al., the lower inter-rater reliability of the results for the 0–3 and 4–7 month groups is due to the fact that younger children are able to perform fewer items than older children, which significantly weakens the ICC value [61].

Our study, similarly to Snyder et al. [49], assessed the agreement in an assessment of motor development between an experienced physiotherapist and a student who received a short theoretical overview. In both studies, similar results for both raters were shown, obtaining a total ICC of 0.99 (our study) and 0.98 (Snyder et al. [49]). Therefore, it can be concluded that the AIMS manual and assessment rules allow for adequate assessment, even when performed by an inexperienced assessor. The pictures and simple descriptions use in the AIMS led students to report improvements in their skills in the area of assessing infant motor development, which underscores that the tool can be used effectively in the classroom.

Assessment of motor development using the AIMS in pediatric patients who change body position frequently and those who are highly sensitive may be a challenge for inexperienced therapists. Some positions in the first months of life, such as standing, may not be achieved by young patients without additional support. It could be difficult for an inexperienced therapist to assess trunk control just by observation without manual assessment. In our study, the greatest difficulty for the inexperienced assessor was assessing lumbar lordosis in two four-point kneeling positions.

It should be noted that a more detailed explanation of the “motor window” used in the AIMS should help in motor development assessment. Also, it is worth expanding the number of items in the standing subscale in the first months of life. We agree with other authors who have recommended further studies comparing the reliability of the AIMS in a group of patients with various disorders.

The main limitations are that this study was conducted in only one center and it involved only one experienced assessor and only one student for whom there was no test–retest assessment analysis.

## 5. Conclusions

The agreement between two raters with different experiences—a last-year physiotherapy student and a pediatric physiotherapist with 10 years of clinical experience in pediatric physiotherapy—in assessing the motor development and motor skills of patients with heart defects aged 0–18 months using the Polish version of the AIMS is satisfactory for both the total and subgroup scores in each age group. Therefore, motor development assessment using the AIMS can be performed on these patients by clinically inexperienced last-year physiotherapy students who are familiarized with the AIMS manual. However, the assessment of test–retest reliability for the AIMS examination performed by clinically inexperienced last-year medical students and/or early graduates on a cohort of pediatric cardiac patients should be performed in future studies. There is a need to update the tool in the supine and prone positions due to doubts about the assessment methodology by clinicians.

## Figures and Tables

**Table 1:** Patients’ characteristics.

	0–3 Months	4–7 Months	8–11 Months	12–18 Months
N	19	23	18	20
Sex (♀/♂)	7/12	12/11	10/8	9/11
Diagnosis (ConHD/cardiac arrhythmia/cardiomyopathy)	19/0/0	21/1/1	16/1/1	19/1/0
Delivery (vaginally/ cesarean)	9/10	11/12	5/13	13/7
Gestational age (weeks)	38 (34–41)	39 (35–41)	38 (27–40)	39 (31–41)
Birth weight (g)	3190 (1630–4380)	3230 (2100–4490)	3115 (840–4120)	3380 (1180–4070)
Apgar score (points)	10 (5–10)	10 (8–10)	10 (5–10)	10 (5–10)

N—number of participants; ♀—females; ♂—males; ConHD—congenital heart disease.

**Table 2:** Inter-rater agreement statistics.

		Experienced Assessor Mean ± SD	Inexperienced Assessor Mean ± SD	ICC (95% CI)	Bias (95% CI)	−1.96 SD (95% CI)	+1.96 SD (95% CI)
0–3 months (n = 19)	Prone	1.74 ± 1.10	1.68 ± 1.06	0.965 (0.910–0.986)	0.05 (−0.14–0.25)	−0.74 (−1.08–−0.40)	0.85 (0.51–1.19)
	Supine	2.42 ± 0.84	2.42 ± 0.84	1.000	0.0 (0.0–0.0)	0.0 (0.0–0.0)	0.0 (0.0–0.0)
	Sitting	0.63 ± 0.68	0.63 ± 0.68	1.000	0.0 (0.0–0.0)	0.0 (0.0–0.0)	0.0 (0.0–0.0)
	Standing	1.26 ± 0.65	1.21 ± 0.54	0.875 (0.682–0.952)	0.05 (−0.14–0.25)	−0.74 (−1.08–−0.40)	0.85 (0.51–1.19)
	Total	6.05 ± 2.74	5.95 ± 2.95	0.989 (0.971–0.996)	0.11 (−0.17–0.38)	−1.01 (−1.48–−0.53)	1.22 (0.74–1.69)
4–7 months (n = 23)	Prone	5.43 ± 3.10	5.35 ± 3.02	0.995 (0.989–0.998)	0.09 (−0.09–0.27)	−0.73 (−1.04–−0.42)	0.90 (0.59–1.22)
	Supine	6.09 ± 2.13	6.26 ± 2.22	0.986 (0.968–0.994)	−0.17 (−0.39–0.04)	−1.14 (−1.51–−0.77)	0.79 (0.42–1.16)
	Sitting	1.78 ± 0.90	1.83 ± 0.98	0.988 (0.971–0.995)	−0.04 (−0.13–0.05)	−0.45 (−0.61–−0.30)	0.37 (0.21–0.52)
	Standing	2.09 ± 0.60	2.13 ± 0.63	0.904 (0.777–0.959)	−0.04 (−0.20–0.12)	−0.76 (−1.04–−0.49)	0.68 (0.40–0.95)
	Total	15.39 ± 5.96	15.57 ± 6.08	0.995 (0.989–0.998)	−0.17 (−0.54–0.19)	−1.81 (−2.43–−1.18)	1.46 (0.84–2.09)
8–11 months (n = 18)	Prone	14.39 ± 5.44	14.06 ± 5.34	0.994 (0.985–0.998)	0.33 (−0.05–0.72)	−1.17 (−1.83–−0.51)	1.84 (1.17–2.50)
	Supine	9.17 ± 3.07	9.11 ± 3.10	0.998 (0.996–0.999)	0.06 (−0.06–0.17)	−0.41 (−0.61–−0.20)	0.52 (0.31–0.72)
	Sitting	9.06 ± 3.61	9.33 ± 3.43	0.992 (0.979–0.997)	−0.28 (−0.56–0.01)	−1.40 (−1.90–−0.91)	0.85 (0.35–1.35)
	Standing	5.56 ± 2.96	5.44 ± 2.87	0.997 (0.991–0.999)	0.11 (−0.05–0.27)	−0.52 (−0.80–−0.24)	0.75 (0.47–1.03)
	Total	38.17 ± 11.59	37.94 ± 11.49	0.998 (0.994–0.999)	0.22 (−0.33–0.78)	−1.96 (−2.93–−0.99)	2.41 (1.44–3.37)
12–18 months (n = 20)	Prone	14.05 ± 6.16	14.05 ± 6.00	0.997 (0.992–0.999)	0.00 (−0.34–0.34)	−1.42 (−2.01–−0.83)	1.42 (0.83–2.01)
	Supine	12.40 ± 5.81	12.35 ± 5.86	0.999 (0.999–0.999)	0.05 (−0.06–0.16)	−0.39 (−0.57–−0.21)	0.49 (0.31–0.67)
	Sitting	10.95 ± 2.61	10.95 ± 2.61	1.000	0.0 (0.0–0.0)	0.0 (0.0–0.0)	0.0 (0.0–0.0)
	Standing	9.30 ± 5.12	9.35 ± 5.11	0.999 (0.999–0.999)	−0.05 (−0.16–0.06)	−0.49 (−0.67–−0.31)	0.39 (0.21–0.57)
	Total	46.70 ± 12.70	46.70 ± 12.61	0.999 (0.997–0.999)	0.00 (−0.40–0.40)	−1.68 (−2.38–−0.98)	1.68 (0.98–2.38)
Total sample (n = 80)	Prone	8.73 ± 6.92	8.61 ± 6.82	0.998 (0.997–0.999)	0.11 (−0.02–0.25)	−1.05 (−1.28–−0.83)	1.28 (1.05–1.51)
	Supine	7.49 ± 5.01	7.51 ± 5.01	0.999 (0.998–0.999)	−0.03 (−0.10–0.05)	−0.65 (−0.77–−0.53)	0.60 (0.48–0.72)
	Sitting	5.44 ± 4.99	5.51 ± 5.00	0.999 (0.998–0.999)	−0.07 (−0.14–−0.01)	−0.68 (−0.80–−0.56)	0.53 (0.41–0.65)
	Standing	4.48 ± 4.32	4.46 ± 4.32	0.999 (0.998–0.999)	0.01 (−0.06–0.09)	−0.65 (−0.78–−0.52)	0.67 (0.55–0.80)
	Total	26.13 ± 18.71	26.10 ± 18.65	0.999 (0.999–0.999)	0.03 (−0.17–0.22)	−1.65 (−1.98–−1.33)	1.70 (1.38–2.03)

SD—standard deviation; ICC—intraclass correlation coefficient; CI—confidence interval.

## Data Availability

Data can be provided by the corresponding author upon reasonable request.
